# White matter integrity in hospitalized COVID-19 patients is not associated with short- and long-term clinical outcomes

**DOI:** 10.3389/fneur.2024.1440294

**Published:** 2024-08-08

**Authors:** Theresa J. van Lith, Hao Li, Marte W. van der Wijk, Naomi T. Wijers, Wouter M. Sluis, Marieke J. H. Wermer, Frank-Erik de Leeuw, Frederick J. A. Meijer, Anil M. Tuladhar

**Affiliations:** ^1^Department of Neurology, Donders Center for Medical Neurosciences, Radboud University Medical Center, Nijmegen, Netherlands; ^2^Department of Neurology, Leiden University Medical CenterLeiden, Netherlands; ^3^Department of Neurology and Neurosurgery, Brain Center, University Medical Center Utrecht, Utrecht, Netherlands; ^4^Department of Neurology, University Medical Center Groningen, Groningen, Netherlands; ^5^Department of Medical Imaging, Radboud University Medical Center, Nijmegen, Netherlands

**Keywords:** COVID-19, white matter integrity, NODDI, DTI, MRI, DWI, SVD

## Abstract

**Objectives:**

SARS-CoV-2 infection is associated with a decline in functional outcomes; many patients experience persistent symptoms, while the underlying pathophysiology remains unclear. This study investigated white matter (WM) integrity on brain MRI in hospitalized COVID-19 patients and its associations with clinical outcomes, including long COVID.

**Materials and methods:**

We included hospitalized COVID-19 patients and controls from CORONavirus and Ischemic Stroke (CORONIS), an observational cohort study, who underwent MRI-DWI imaging at baseline shortly after discharge (<3 months after positive PCR) and 3 months after baseline scanning. We assessed WM integrity using diffusion tensor imaging (DTI) and neurite orientation dispersion and density imaging (NODDI) and performed comparisons between groups and within patients. Clinical assessment was conducted at 3 and 12 months with functional outcomes such as modified Rankin Scale (mRS), Post-COVID-19 Functional Status scale (PCFS), Visual Analogue Scale (VAS), and long COVID, cognitive assessment was conducted by the Modified Telephone Interview for Cognitive Status (TICS-M), and the Hospital Anxiety and Depression Scale (HADS) was used to assess mood disorder. Associations between WM integrity and clinical outcomes were evaluated using logistic regression and linear regression.

**Results:**

A total of 49 patients (mean age 59.5 years) showed higher overall peak width of skeletonized mean diffusivity (PSMD) (*p* = 0.030) and lower neurite density index (NDI) in several WM regions compared with 25 controls at the baseline (*p* < 0.05; FWE-corrected) but did not remain statistically significant after adjusting for WM hyperintensities. Orientation dispersion index (ODI) increased after 3-month follow-up in several WM regions within patients (*p* < 0.05), which remained significant after correction for changes in WMH volume. Patients exhibited worse clinical outcomes compared with controls. Low NDI at baseline was associated with worse performance on the Post-COVID-19 Functional Status scale after 12 months (*p* = 0.018).

**Conclusion:**

After adjusting for WMH, hospitalized COVID-19 patients no longer exhibited lower WM integrity compared with controls. WM integrity was generally not associated with clinical assessments as measured shortly after discharge, suggesting that factors other than underlying WM integrity play a role in worse clinical outcomes or long COVID.

## 1 Introduction

Coronavirus disease 2019 (COVID-19) is associated with a wide range of symptoms (1–3). Many patients experience reduced functional performance and persistent symptoms several months after infection, including fatigue, cognitive impairment, mood disorders, and anosmia, often referred to as long COVID (4). However, the underlying pathophysiological mechanism for these ongoing symptoms remains unknown. Previous studies investigating the white matter (WM) microstructure with diffusion-weighted imaging (DWI) in COVID-19 patients, conducting scans from several months to 2 years after discharge, yielded conflicting results (5–7). Two studies, with scans conducted for several months after discharge, reported an association between lower WM integrity in several brain regions and a decline in cognition (6, 7). Meanwhile, a long-term follow-up study reported WM integrity recovery (or increased) 2 years after COVID-19 infection in comparison to measurements conducted 1 year after infection (6–8). However, it is unknown whether these changes in alterations in WM integrity reflect COVID-19 pathology or age-related processes as previous studies performed baseline and follow-up MRI from several months to years after the acute, inflammatory symptomatic phase of the infection. In addition, previous studies did not consider comorbidity with pre-existing brain damage, such as white matter hyperintensities (WMH) due to underlying cerebral small vessel disease (SVD), which is highly associated with decreased WM integrity (9). Finally, previous studies mainly focused on cognition, but the relation with functional outcomes such as mood disorder, or long COVID has not yet been investigated.

Our aim was to investigate WM integrity using diffusion metrics, derived from both diffusion tensor imaging (DTI) and neurite orientation dispersion and density imaging (NODDI) models, in patients hospitalized for COVID-19 compared with unaffected controls (with no signs of previous SARS-CoV-2 infection in laboratory results) while taking into account the presence of WMH. In addition, we examined changes in WM integrity in patients over 3 months and, finally, investigated the relation between diffusion metrics and short- and long-term clinical outcomes (3 and 12 months after discharge).

## 2 Materials and methods

### 2.1 Participants

This study is part of the CORONavirus and Ischemic Stroke (CORONIS) study, a multicenter prospective observational cohort study in the Netherlands examining MRI markers of cerebrovascular disease in patients hospitalized for COVID-19. The study aimed to prospectively perform brain MRI in unselected hospitalized COVID-19 patients, who were not exhibiting neurological symptoms. A detailed description of the study protocol has been described elsewhere (10). Participants (*n* = 202) were recruited in three academic hospitals between April 2021 and October 2022. The inclusion criteria for patients are (a) hospitalization due to COVID-19, (b) PCR-confirmed diagnosis, and (c) ≥18 years old. Healthy controls, age- and sex-matched, were recruited from patients’ relatives or through the hospital, requiring a negative anti-Sars-CoV-2 IgG test at inclusion. The exclusion criteria for both groups were MRI contra-indications, pregnancy, or limited life expectancy (<3 months). Additionally, to address potential confounders, we excluded patients with conditions such as cardiac arrest or PRES, which were not present in our cohort. For this study, we only included participants from a single site (Radboudumc) who underwent multi-shell DWI, which was not conducted in the other participating centers. COVID-19 patients with overt ischemic stroke were excluded. This study was approved by the Medical Review Ethics Committee of Arnhem-Nijmegen on 1 April 2021. All participants provided written informed consent.

### 2.2 Data collection

Baseline data collection included demographics, lifestyle, education, medical history, and hospitalization details (10). Brain MRI scans were performed at baseline; for COVID-19 patients, either during admission or shortly after discharge within 3 months following a positive PCR, and for healthy controls, they signed the informed consent. Follow-up MRIs were performed for COVID-19 patients after 3 months. Healthy controls did not undergo a follow-up MRI. Telephone follow-up questionnaires were collected at 3 and 12 months after the inclusion of all participants (for COVID-19 patients during a 3-month MRI visit). Education levels were categorized on a validated scale from 1 (below primary school) to 7 (academic degree) and grouped into ‘low’ (levels 1–3), ‘middle’ (level 4), and ‘high’ (above level 4) following the Verhage scaling system (11). The type of ventilation was classified into three categories to indicate the severity of COVID-19: (1) non-invasive respiratory support, including nasal cannula or non-rebreathing mask; (2) non-invasive ventilation, such as Optiflow; and (3) invasive ventilation, involving intubation.

### 2.3 Neuroimaging protocol

All participants underwent the same scanning protocol on a 3 Tesla MRI scanner (Siemens Prisma) at baseline and during follow-up. The protocol included the following sequences: 3D T1 weighted (T1W) space fatsat with the following parameters: 0.9 ms isotropic voxel size, repetition time (TR) = 700 ms, and echo time (TE) = 9 mm; 3D fluid-attenuated inversion recovery (FLAIR) with the following parameters: 1 mm isotropic voxel size and TR = 500 ms and TE = 394 ms; multi-shell DWI with the following parameters: 80 diffusion-weighted directions (40 × b = 1,000, and 40 × b = 2,000 s/mm2), 6 × b = 0 images, 2.0 mm isotropic voxels, and TR = 4,600 ms and TE = 80 ms (10). Imaging details have been described previously (10).

### 2.4 White matter hyperintensity (WMH) volume

We used a validated segmentation method based on the k-nearest neighbor algorithm (UBO detector) to automatically segment WMH and calculate WMH volumes using bias-corrected T1 and FLAIR images (12). Segmentations were visually reviewed for errors or artifacts. Note that the UBO detector calculated WMH volumes in SPM’s DARTEL space. Therefore, there is no need to adjust for intracranial volume.

### 2.5 Diffusion MRI preprocessing and metrics

Diffusion MRI data were pre-processed to remove the noise and Gibbs artifacts, correct head motion, Eddy current-induced distortion, susceptibility-induced distortion (top-up), and intensity bias using MRtrix 3.0 software (13), Functional Magnetic Resonance Imaging of the Brain Software Library (FSL) software (14), and advanced normalization tools (5). Due to the absence of a b0 image with reversed phase encoding in our DWI scans, ‘topup’ was performed based on a synthesized b0 image from the T1 image using Synb0-DISCO (15). Diffusion metrics derived from different diffusion models (DTI and NODDI) were calculated. While DTI-derived measures [including fractional anisotropy (FA) and mean diffusivity (MD)] have been widely used, they only provide a composite view of contributions from multiple tissue components [intra-neurite, extra-neurite, and cerebral spinal fluid (CSF)] within the voxel. In contrast, the NODDI model can delineate contributions from each compartment, offering measures such as neurite density index (NDI), orientation dispersion index (ODI), and cerebrospinal fluid volume fraction (fCSF) of each tissue component within one voxel (6). First, two DTI metrics were calculated with the pre-processed diffusion data (only *b* = 0 and *b* = 1,000): mean diffusivity (MD) and fractional anisotropy (FA) maps of each participant using the ‘dtifit’ function within FSL (16). Second, we used the entire multi-shell DWI data to fit the NODDI model using the NODDI toolbox in MATLAB (http://mig.cs.ucl.ac.uk/index.php?n=Tutorial.NODDImatlab). Using this tool, we calculated three NODDI parameters: NDI, ODI, and fCSF maps for each participant (17). Peak width of Skeletonized Mean Diffusivity (PSMD) values were calculated using the PSMD tool (http://www.psmd-marker.com/) (16).

### 2.6 Tract-based spatial statistics

Voxel-wise statistical analysis of FA maps was conducted using the Tract-Based Spatial Statistics (TBSS) pipeline (18), which is a component of FSL software (version 6.0.1) (14, 18, 19). FA maps from all participants (including scans of patients at baseline and follow-up and healthy controls) were fed into the TBSS pipeline tool to create a mean FA skeleton representing the centers of all tracts shared across the population. Next, MD, NDI, ODI, and fCSF maps were projected into the WM skeleton using the ‘tbss_non_FA’ function within the TBSS tool of FSL, resulting in aligned maps of FA, MD, NDI, ODI, and fCS. Finally, the resultant five maps were analyzed using voxel-wise cross-subject statistics.

### 2.7 Clinical outcomes during follow-up

Functional outcomes included the modified Rankin Scale (mRS), Post-COVID-19 Functional Status (PCFS) scale, visual analog scale (VAS), and long COVID [defined following the WHO definition and Delphi (2021) consensus] (20–23). Symptoms reported by patients were collected during the two follow-up stages (physical and telephone interviews). Since participants were explicitly asked for fatigue and dyspnea, we only adjudicated these symptoms to long COVID if they had an impact on everyday functioning. This was expressed as a decline on the PCFS scale between study procedures (baseline and 3-month and 12-month follow-up). The cognitive assessment included the Modified Telephone Interview for Cognitive Status (TICS-M). To assess symptoms of anxiety and depression (mood), the Hospital Anxiety and Depression Scale (HADS) was used (24).

### 2.8 Statistical analysis

Baseline characteristics of patients and controls and clinical outcomes were compared using chi-square tests for categorical variables (*n*, %) and the *t*-test (mean, SD) or Wilcoxon rank-sum (median, IQR) tests for continuous data. mRS and PCFS were categorized in the same two groups (0–1, good vs. ≥2, poor outcome). For the cross-sectional comparison (patients vs. controls) of diffusion metrics (PSMD and WMH volume), linear regression adjusted for age and sex was performed. Voxel-wise group comparisons of FA, MD, NDI, ODI, and fCSF maps between (1) patients and controls [adjusted for age and sex (model 1) and additionally for WMH volume (model 2)] and (2) within patients (baseline vs. follow-up) were performed using the FSL randomize tool (permutation-based inference: 5,000 permutations). Significant clusters were identified using threshold-free cluster enhancement-based family-wise error correction for multiple comparisons in TBSS analyses (*p* < 0.05) (25). Longitudinal analysis of clinical outcomes within patients included McNemar’s test (*n*, %) for categorical variables and two-sample t-tests and Wilcoxon tests (non-normal distributions) for continuous data. DTI and NODDI metrics values of regions that differed between groups were extracted. Logistic and linear regression were used to analyze the relationship between significant MRI parameters (PSMD and NDI) and clinical outcomes, reported as crude odds ratios (OR) and adjusted odds ratios (aOR) (95% CIs) corrected for age (model 1) and additionally WMH (model 2) and multiple testing using false discovery rate (FDR) (26). Subgroup analyses of diffusion metrics were performed between (a) ICU and non-ICU patients and (b) patients with long COVID and patients without long COVID. Education was grouped into three levels for the comparison of baseline characteristics and the original seven levels for logistic and linear regression. Two-sided *p*-values of <0.05 were considered statistically significant. Data were analyzed using R version 4.3.1.

## 3 Results

### 3.1 Participants

**Figure 1:**
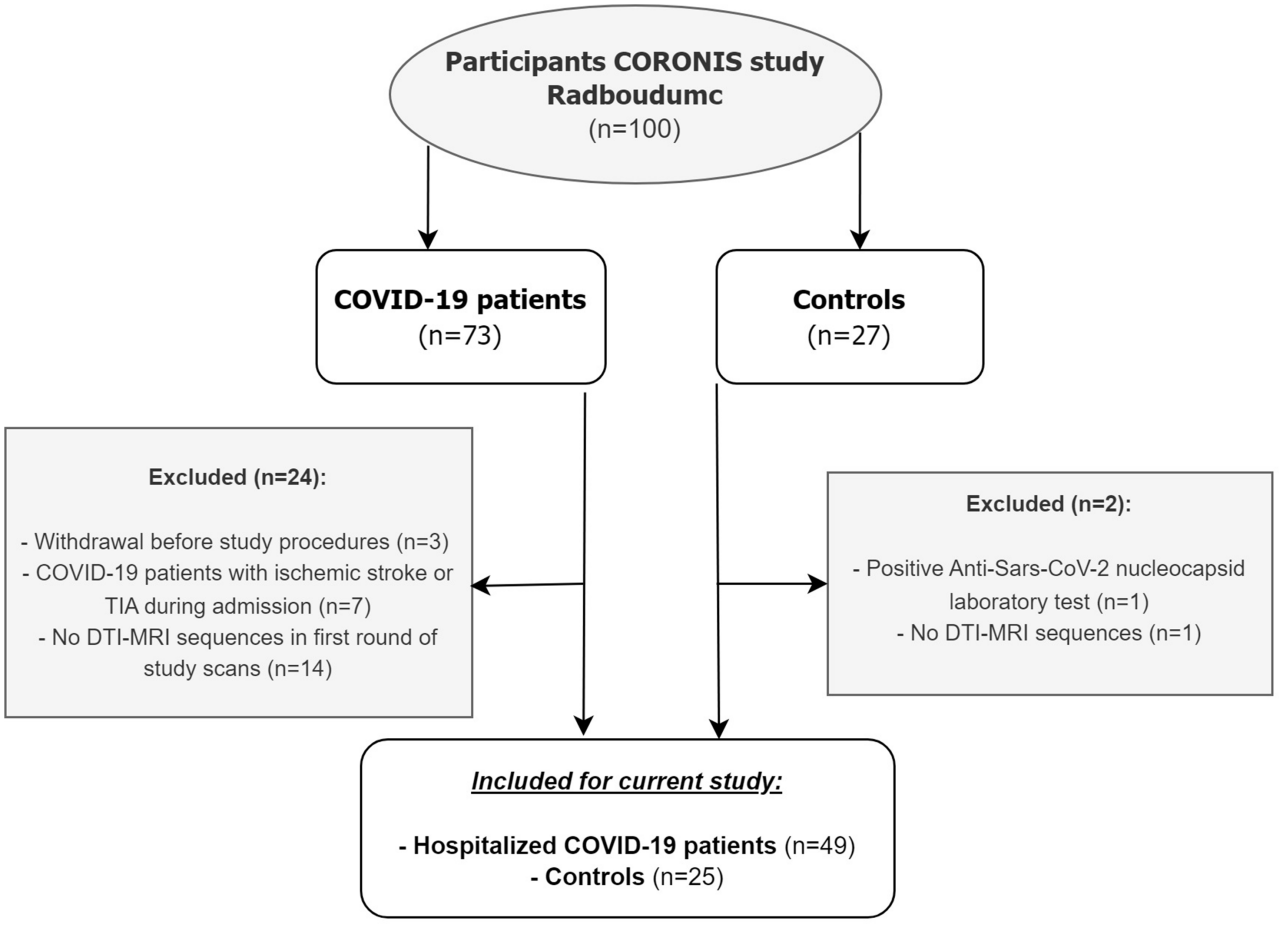
Flowchart of study population. TIA = transient ischemic attack, DTI = diffusion tensor imaging.

In total, 73 patients and 27 controls were eligible for participation, of whom 24 patients and 2 controls were excluded (Figure 1). This study resulted in 49 patients with COVID-19 [mean age: 59.5 years (SD 12.6); 32.7% female, 30.6% admitted to ICU] and 25 healthy controls [mean age: 58.5 years (SD 10.1); 48.0% female]. Baseline characteristics are shown in Table 1. There were no differences between patients and controls regarding age or sex. Regarding all participants, the time between baseline assessments and follow-up 1 was 111 days [median, IQR (93.0–140.0)] and between follow-up 1 and follow-up 2 was 249 days [IQR 224.0–271.0] (+ − 8.2 months). Radiologists have reviewed the brain MRI for the presence of acute disseminated encephalomyelitis (ADEM), acute necrotizing encephalitis, posterior reversible encephalopathy syndrome (PRES), and osmotic demyelination syndrome, as these conditions could influence the white matter abnormalities, and they were not observed in our study population.

**Table 1:** Baseline characteristics COVID-19 patients vs. controls.

	**COVID-19 patients** ***n* = 49**	**Controls** ***n* = 25**	*p*-value*
Female, *n* (%)	16 (32.7)	12 (48.0)	0.301
Age at inclusion, mean (SD)	59.53 (12.63)	58.48 (10.06)	0.719
COVID vaccine before admission/inclusion, *n* (%)	21 (42.9)	22 (88.0)	**0.011**
ICU admission, *n* (%)	15 (30.6)	N/A	N/A
**Respiratory or ventilation therapy required at maximum during admission, *n* (%)**			
Nasal cannula, oxygen mask, non-rebreathing mask	26 (53.2)	N/A	N/A
Non-invasive ventilation (Optiflow)	15 (30.5)		
Invasive ventilation (intubation)	8 (16.3)		
Days hospital admission, median [IQR]	11.0 [7.0, 15.0]	N/A	N/A
Days between positive PCR and baseline MRI, median [IQR]	40.0 [30.0, 54.0]	N/A	N/A
Days between positive PCR and inclusion study, median [IQR]	16.0 [8.0, 27.0]	N/A	N/A
Days between hospital admission and baseline MRI, median [IQR]	32.00 [27.0–48.0]	N/A	N/A
Education level, *n* (%)			0.085
Low	8 (16.3)	6 (24.0)	
Middle	27 (55.1)	7 (28.0)	
High	14 (28.6)	12 (48.0)	
**Cardiovascular history**			
Body Mass Index (BMI) in kg/m^2^, mean (SD)	28.40 (4.31)	27.46(3.35)	0.344
Smoking, *n* (%)	29 (59.2)	11 (44.0)	0.321
Diabetes Mellitus, *n* (%)	10 (20.4)	2 (8.0)	0.300
Hypertension, *n* (%)	19 (38.8)	3 (12.0)	**0.034**
Hypercholesterolemia, *n* (%)	21 (42.9)	6 (24.0)	0.181
Pulmonary disease (e.g., COPD, asthma), *n* (%)	21 (42.9)	1 (4.0)	**0.001**
**MRI characteristics**			
White Matter Hyperintensity (WMH) volume (mm^3^), median [IQR]	1.481 [0.884–3.435]	0.857 [0.392–1.512]	**0.021**
Microbleeds, *n* (%)	5 (10.2)	2 (8.0)	0.759

*Unadjusted *p*-values.

ICU = intensive care unit, PCR = polymerase chain reaction, COPD = chronic obstructive pulmonary disease, N/A = not applicable.The bold values indicate significance (*p* < 0.05).

### 3.2 WM integrity—cross-sectional analyses

**Figure 2:**
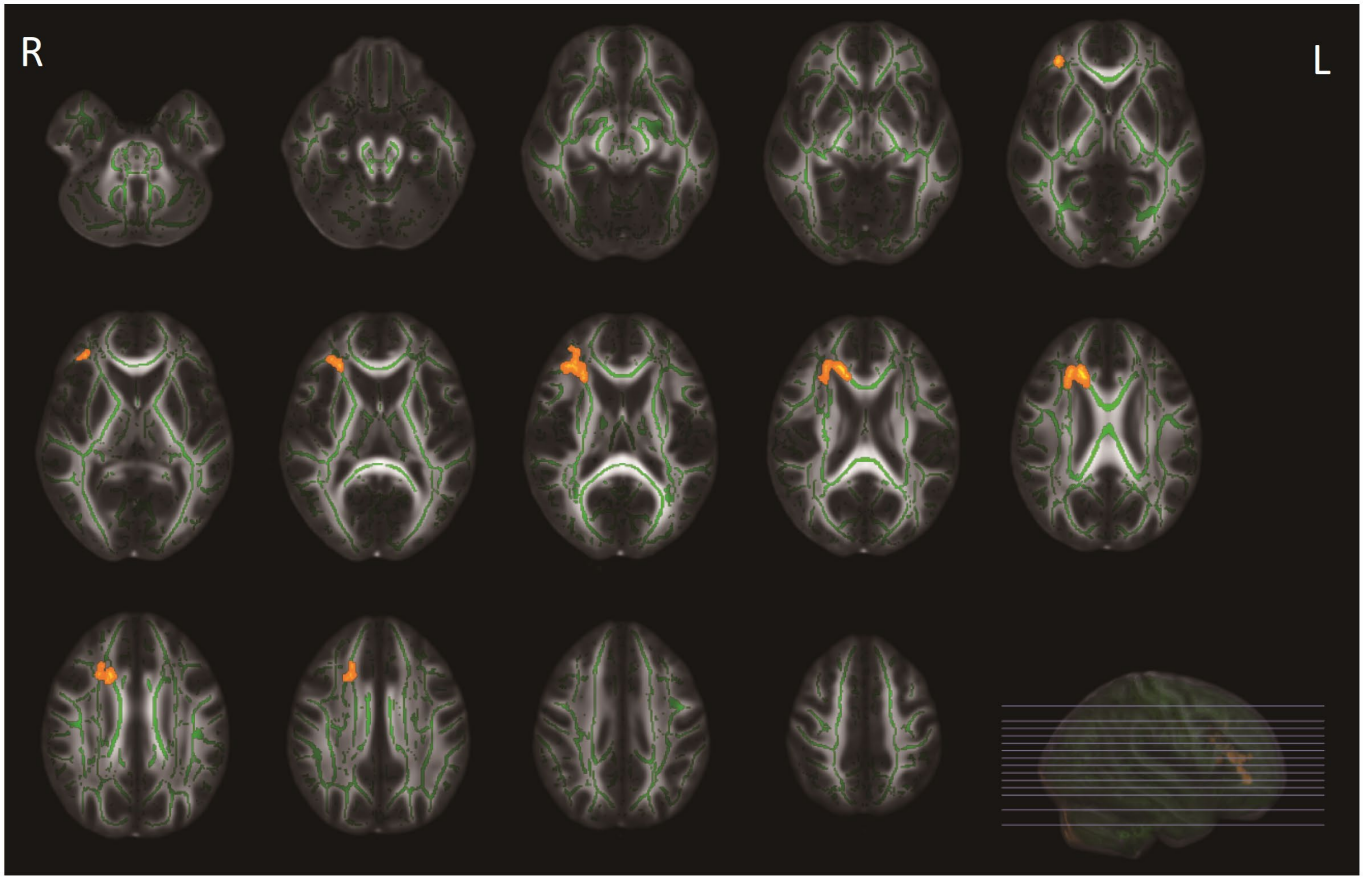
Tract-based spatial statistics (TBSS) analyses of neurite density index (NDI) values between patients with COVID-19 and healthy controls. WM tracts in orange represent regions with significantly lower NDI values in patients with COVID-19 compared with healthy controls, after adjusting for age and sex, and for multiple comparisons (*p* < 0.05, family-wise error corrected). These differences disappeared after additionally adjusting for WMH volumes.

At baseline, patients with COVID-19 exhibited higher age- and sex-adjusted PSMD values (mean = 1.70 *10^−4^ mm^2^/s, SD = 0.29 *10^−4^ mm^2^/s) than controls (mean = 1.56 *10^−4^ mm^2^/s, SD = 0.13*10^−4^ mm^2^/s) *(p = 0.030)*. After additional correction for WMH volume, this difference was not statistically significant (*p = 0.590*). There were no significant differences in FA and MD values using voxel-wise analyses between groups (*p*-corrected values >0.05). Compared with controls, adjusted for age and sex, patients demonstrated significantly lower NDI values in the right anterior thalamic radiation (ATR), forceps minor, and right inferior fronto-occipital fasciculus (Figure 2). These differences disappeared after additionally adjusting for WMH volumes. Voxel-based analyses showed no significant differences in ODI and fCSF between patients and controls. In a subgroup analysis, there were no significant differences in diffusion metrics between COVID-19 patients with long COVID and COVID-19 patients without long COVID. Additionally, no significant differences were found in diffusion metrics between ICU patients and non-ICU patients.

### 3.3 Changes in WM integrity in patients over time

**Figure 3:**
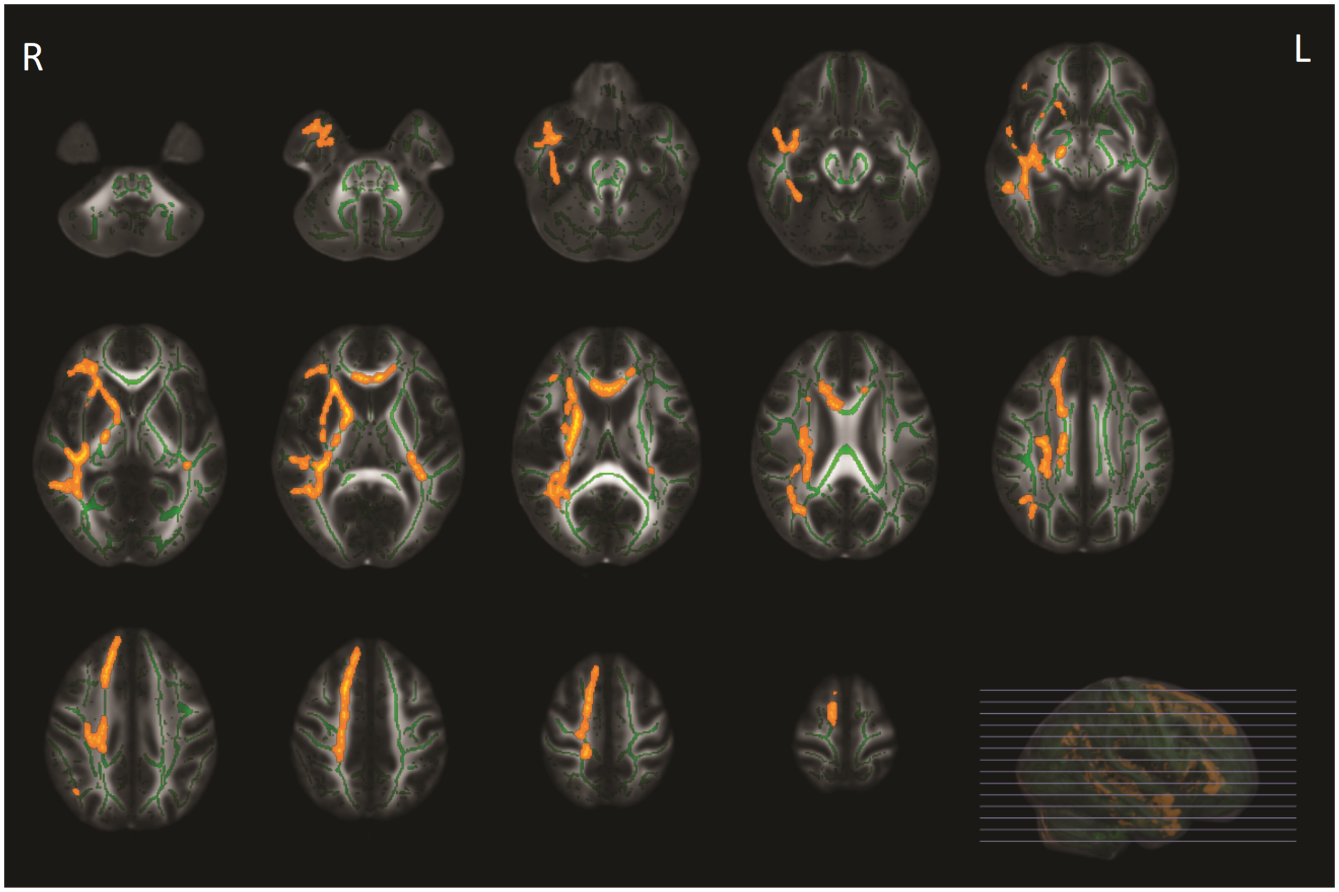
Tract-based spatial statistics (TBSS) analyses of orientation dispersion index (ODI) values in patients with COVID-19 between baseline and follow-up MRI. WM tracts in yellow-orange represent regions with significant ODI values in COVID-19 at baseline compared with ODI values after 3-month follow-up, adjusted for multiple comparisons (*p* < 0.05, family-wise error corrected).

Of the 49 patients at baseline, 39 (79.6%) patients underwent brain MRI during follow-up after 3 months. Ten patients were excluded because they did not undergo follow-up MRI due to various reasons [illness (*n* = 1), lost to follow-up (*n* = 5), claustrophobia (*n* = 3), and moved to a foreign country (*n* = 1)]. ODI values increased after 3 months of follow-up in patients compared with baseline in the ATR, bilateral corticospinal tract, cingulum (cingulate gyrus and hippocampus), forceps major and minor, inferior fronto-occipital fasciculus on both sides, bilateral inferior fronto-occipital fasciculus left, superior longitudinal fasciculus left and right, and the right uncinate fasciculus (Figure 3), which remained significant after correction for the change of WMH volume. However, other diffusion metrics and WMH volumes did not change over time.

### 3.4 Clinical outcomes during follow-up

COVID-19 patients had worse functional outcomes compared with controls 3 and 12 months after discharge. Patients had more symptoms of depression (*p* = 0.008), lower scores on cognitive functions, as measured by TICS-M at 12 months, and lower scores on the VAS scale (*p* = 0.006) (Table 2). Long COVID was present in 62.2% of the patients after 3 months and in 40.9% after 12 months. Apart from a decrease in frequency of long COVID, no differences in functional and cognitive outcomes were detected in patients between the 3- and 12-month follow-up (Supplementary Table S2).

**Table 2:** Functional, cognitive outcome and mood symptoms between groups.

**Outcomes**		**COVID-19 patients**		**Controls**	
	*n* total		*n* total		*p*-value*
**3-month follow-up**					
**Cognitive function**					
TICS-M score, mean (SD)	45	36.22 (4.16)	20	37.20 (3.09)	0.386
**Functional outcomes**					
mRS (reference 0–1) ≥2–6, *n* (%)	45	15 (33.3)	20	0 (0.0)	**0.006**
PCFS (reference 0–1) ≥2–4, *n* (%)	45	22 (48.9)	20	2 (10.0)	**0.006**
**Mood**					
*Hospital Anxiety and Depression Scale*					
HADS-Anxiety, median [IQR]	45	3.0 [1.0, 6.0]	20	3.0 [0.8, 5.5]	0.474
HADS-Depression, median [IQR]	45	2.0 [1.0, 7.0]	20	1.0 [0.0, 2.0]	0.095
**Long COVID** ^**a**^ **, *n* (%)**	45	28 (62.2)	–	–	–
**12-month follow-up**					
**Cognitive function**					
TICS-M, mean (SD)	42	35.24 (4.66)	21	38.81 (3.60)	**0.006**
**Functional outcomes**					
mRS (reference 0–1) ≥2–6, *n* (%)	44	16 (36.4)	22	0 (0.0)	**0.004**
PCFS (reference 0–1) ≥2–4, *n* (%)	44	22 (50.0)	22	0 (0.0)	**<0.001**
VAS scale (0–100), mean (SD)	44	73.77 (16.41)	24	85.92 (8.73)	**0.004**
**Mood**					
*Hospital Anxiety and Depression Scale (HADS)*					
HADS-Anxiety, median [IQR]	44	3.5 [0.0, 7.0]	24	2.5 [0.0, 4.0]	0.147
HADS-Depression, median [IQR]	44	3.5 [1.0, 8.0]	24	1.0 [0.0, 2.0]	**0.008**
**Long COVID** ^**a**^ **, *n* (%)**	44	18 (40.9)	–	–	–

*Adjusted for multiple comparisons using false discovery rate (FDR).

^a^Description symptoms reported in Supplementary Table S1.

TICS-M = modified telephone interview for cognitive status, mRS = modified Rankin Scale, PCFS = Post-COVID functional scale, VAS = Visual Analogue Scale, HADS = Hospital Anxiety and Depression Scale, N/A = not applicable.The bold values indicate significance (*p* < 0.05).

### 3.5 Association between WM integrity and clinical outcomes

The diffusion metrics at baseline and the changes in ODI values during follow-up were not related to the clinical outcomes (Supplementary Tables S4–S6). However, lower NDI values of the regions that differed between patients and controls at baseline were significantly associated with lower scores on the PCFS scale (functional outcome) at the 12-month follow-up (Supplementary Table S3), which remained significant after additional adjustment for WMH volumes (*p* = 0.018).

## 4 Discussion

In our study, we showed that after adjusting for WMH volume, hospitalized COVID-19 patients no longer exhibited higher PSMD and lower NDI values compared with controls. Our longitudinal study revealed decreased ODI in several regions of the WM in patients 3 months after COVID hospitalization. Patients exhibited worse clinical outcomes compared with controls after infection but only decreased NDI at baseline was associated with worse performance on the Post-COVID-19 Functional Status scale after 12 months. In addition, we found no associations between diffusion metrics and long COVID. Our results suggest that other factors play a role in poorer clinical outcomes and long COVID in patients several months after COVID-19 infection.

We found lower WM integrity (indicated by higher values of PSMD and lower values of NDI) in patients compared with controls, which disappeared after adjusting for WMH. Several previous studies demonstrated that COVID-19 patients have alterations of the cerebral WM identified by DWI which are present 1 year after infection (27, 28). Some of these changes in WM integrity have been attributed to the SARS-CoV-2 infection. COVID-19 could potentially contribute to an increase in WM damage (loss of WM integrity and WMH), as microvascular pathology has been observed with the evidence of infected brain endothelial cells in the histopathology of the brains of COVID-19 patients (29). However, due to the absence of MRI scans conducted before infection in these studies, as well as in our study, it is not possible to demonstrate or rule out the presence of pre-existing WM damage, for which these previous studies also did not correct in analysis. In our opinion, the alterations of the WM found in our study are more likely to be attributed to underlying WMH, since the differences disappeared after correction of this confounder in our study. Patients with COVID-19 had higher WMH volume at baseline, a hallmark of SVD, and were more frequently diagnosed with hypertension compared with the controls. Patients with cardiovascular risk factors, such as hypertension, are at an increased risk of admission for COVID-19 (2). This may explain the higher prevalence of hypertension, SVD, and higher WMH volume in hospitalized COVID-19 patients compared with controls. Therefore, the observed differences are more likely to be explained by the presence of SVD and less likely by the SARS-CoV-2 infection itself.

We found an increase in ODI over time within patients. Lower ODI values in the cerebral WM indicate that the fibers are less dispersed within this voxel, which, in most cases, suggests higher WM microstructural integrity (17). These findings of an increase in ODI might therefore be indicative of ongoing WM loss, which is not captured by other diffusion metrics. This finding, however, contradicts the results of an earlier study that examined COVID-19 patients 1 and 2 years after their discharge (7, 8). This study reported a decrease in ODI at the 2-year follow-up, suggesting a recovery of the WM over time. The observed discrepancies in results could possibly be attributed to different phases of the SARS-CoV-2 infection—MRI interval. Due to the lack of follow-up MRI in the control group, we were unable to confirm whether this finding could be considered as a deterioration specific to SARS-CoV-2 infection or might be apparent as a “normal” process in the brain. We acknowledge the potential influence of factors such as metabolic syndrome in our results, given the well-known associations between hypertension, hypercholesterolemia, and SVD which are related to WM integrity. This underscores the importance of considering these factors (such as metabolic syndrome) but also other factors such as asthma and COPD (beyond the scope of our current study) in future research.

Our study revealed that patients hospitalized for COVID-19 exhibited worse clinical outcomes during follow-up assessments after 3 and 12 months compared with the control group. Here, we only found an association between lower NDI values (in the MRI shortly after discharge) and worsened PCFS (global functional outcome scale) at 12 months. In contrast to previous studies on DTI and clinical outcomes, we found no associations with cognitive scores (7, 8). First, this could be due to the fact that the cognitive assessment tool we used (TICS-m) may lack an in-depth evaluation of cognitive function. Second, the relatively small sample size may have led to a type II error. Finally, the relative WMH damage to regions with abnormal NDI values might not have been enough to cause a noticeable, symptomatic decline in cognitive behavior or other clinical outcomes during follow-up.

We observed reduced white matter integrity at baseline, indicated by lower NDI, in the anterior thalamic radiation (ATR), forceps minor, and right inferior fronto-occipital fasciculus (IFOF) of patients. In addition, we found a significant association between the lower NDI of these regions and PCFS. Previous research has linked the ATR and forceps minor regions to executive function and processing speed (2). In addition, WMH and reduced white matter integrity in ATR and IFOF were also associated with reduced processing speed (3). Executive function and processing speed can influence overall functioning, for example, possibly the PCFS; however, this relationship requires more thorough investigation using more extensive cognitive assessments.We observed no difference in WM integrity diffusion metrics between patients with long COVID and without long COVID and did not find associations between baseline WM integrity and long COVID during the follow-up. To date, no studies have explored this aspect; however, we hypothesize that our sample size may have been too small to detect a significant difference. Additionally, we did not perform MRI scans after 12 months, on which we determined the frequency of long COVID, which would have been particularly beneficial in this context. Given the lack of a clear association between clinical outcomes and diffusion metrics in a group with a high frequency of poor clinical outcomes, it is likely that factors such as respiratory problems at disease onset, length of hospital stay, and ICU admission may play a more significant role in explaining the clinical status, including long COVID.

There are some limitations that need to be addressed. First, in patients, we performed baseline and post-discharge brain MRI after 3 months. Considering the time in which WM damage arises, including loss of microstructural WM integrity and WMH, a 3-month follow-up period may be too short to capture changes, and it would have been valuable to include a follow-up MRI 1 year after infection for analysis and comparison with clinical outcomes. Second, our sample was relatively small, consisting of hospitalized patients varying from short stay to ICU admission including a small control group. Moreover, the smaller sample size that underwent follow-up MRI may further limit our ability to identify significant associations. Third, patients had not undergone brain MRI before COVID-19, making it impossible to adjudicate WM integrity assessed after the SARS-CoV-2 infection and establish the relationship with the actual infection. Fourth, the cognitive examination was limited. Extended cognitive evaluation may have an added value to identify impairment in specific cognitive domains or more subtle cognitive problems. Fifth, no follow-up imaging was performed in the control group, which limits our comparability of the WM integrity with the patients.

## 5 Conclusion

To conclude, our study revealed lower WM integrity in patients hospitalized for COVID-19 compared with healthy controls, which is likely explained by the presence of WMH and not by SARS-CoV-2 infection itself. After 3 months, we found deterioration of WM integrity within patients. WM integrity at baseline, or the changes after 3 months thereof, was generally not associated with poorer clinical outcomes after 1 year, suggesting that other factors play a more important role in the clinical outcomes and long COVID in patients after SARS-CoV-2 infection.

## Data availability statement

The raw data supporting the conclusions of this article will be made available by the authors, without undue reservation after permission of the local ethics committee.

## Ethics statement

This study was approved by the Medical Review Ethics Committee region Arnhem-Nijmegen on 01 April 2021 (NL75780.091.20). The studies were conducted in accordance with the local legislation and institutional requirements. The participants provided their written informed consent to participate in this study.

## Author contributions

TL: Conceptualization, Data curation, Formal analysis, Investigation, Methodology, Visualization, Writing – original draft, Writing – review & editing. HL: Formal analysis, Validation, Visualization, Writing – review & editing. MWW: Data curation, Writing – review & editing. NW: Writing – review & editing. WS: Writing – review & editing. MJW: Funding acquisition, Writing – review & editing. F-EL: Conceptualization, Funding acquisition, Investigation, Methodology, Project administration, Supervision, Writing – review & editing. FM: Conceptualization, Data curation, Methodology, Visualization, Writing – review & editing. AT: Conceptualization, Formal analysis, Funding acquisition, Methodology, Supervision, Writing – review & editing.

## References

[1] Nasserie T Hittle M Goodman SN. Assessment of the frequency and variety of persistent symptoms among patients with COVID-19: a systematic review. JAMA Netw Open. (2021) 4:e2111417. doi: 10.1001/jamanetworkopen.2021.11417, PMID: 34037731 PMC8155823

[2] Ballering AV SKR VZ Olde Hartman TC JGM R Lifelines Corona Research I. Persistence of somatic symptoms after COVID-19 in the Netherlands: an observational cohort study. Lancet. (2022) 400:452–61. doi: 10.1016/S0140-6736(22)01214-4 35934007 PMC9352274

[3] Taquet M Geddes JR Husain M Luciano S Harrison PJ. 6-month neurological and psychiatric outcomes in 236 379 survivors of COVID-19: a retrospective cohort study using electronic health records. Lancet Psychiatry. (2021) 8:416–27. doi: 10.1016/S2215-0366(21)00084-5, PMID: 33836148 PMC8023694

[4] Huang C Huang L Wang Y Li X Ren L Gu X. 6-month consequences of COVID-19 in patients discharged from hospital: a cohort study. Lancet. (2021) 397:220–32. doi: 10.1016/S0140-6736(20)32656-8, PMID: 33428867 PMC7833295

[5] Avants BB Tustison NJ Song G Cook PA Klein A Gee JC. A reproducible evaluation of ANTs similarity metric performance in brain image registration. NeuroImage. (2011) 54:2033–44. doi: 10.1016/j.neuroimage.2010.09.025, PMID: 20851191 PMC3065962

[6] Lu Y Li X Geng D Mei N Wu PY Huang CC. Cerebral Micro-structural changes in COVID-19 patients - an MRI-based 3-month follow-up study. EClinicalMedicine. (2020) 25:100484. doi: 10.1016/j.eclinm.2020.100484, PMID: 32838240 PMC7396952

[7] Huang S Zhou Z Yang D Zhao W Zeng M Xie X. Persistent white matter changes in recovered COVID-19 patients at the 1-year follow-up. Brain. (2022) 145:1830–8. doi: 10.1093/brain/awab435, PMID: 34918020 PMC8754808

[8] Huang S Zhou X Zhao W Du Y Yang D Huang Y. Dynamic white matter changes in recovered COVID-19 patients: a two-year follow-up study. Theranostics. (2023) 13:724–35. doi: 10.7150/thno.79902, PMID: 36632218 PMC9830428

[9] Ter Telgte A van Leijsen EMC Wiegertjes K Klijn CJM Tuladhar AM de Leeuw FE. Cerebral small vessel disease: from a focal to a global perspective. Nat Rev Neurol. (2018) 14:387–98. doi: 10.1038/s41582-018-0014-y, PMID: 29802354

[10] van Lith TJ Sluis WM Wijers NT Meijer FJ Kamphuis-van Ulzen K de Bresser J. Prevalence, risk factors, and long-term outcomes of cerebral ischemia in hospitalized COVID-19 patients - study rationale and protocol of the CORONIS study: a multicentre prospective cohort study. Eur Stroke J. (2022) 7:180–7. doi: 10.1177/23969873221092538, PMID: 35647315 PMC9134783

[11] Verhage F. Intelligentie en leeftijd; onderzoek bij Nederlandser van twaalf tot zevenenzeventig jaar. Assen: Van Gorcum (1964).

[12] Jiang J Liu T Zhu W Koncz R Liu H Lee T. UBO detector - a cluster-based, fully automated pipeline for extracting white matter hyperintensities. NeuroImage. (2018) 174:539–49. doi: 10.1016/j.neuroimage.2018.03.050, PMID: 29578029

[13] Tournier JD Smith R Raffelt D Tabbara R Dhollander T Pietsch M. MRtrix3: a fast, flexible and open software framework for medical image processing and visualisation. NeuroImage. (2019) 202:116137. doi: 10.1016/j.neuroimage.2019.116137, PMID: 31473352

[14] Smith SM Jenkinson M Woolrich MW Beckmann CF Behrens TE Johansen-Berg H. Advances in functional and structural MR image analysis and implementation as FSL. NeuroImage. (2004) 23:S208–19. doi: 10.1016/j.neuroimage.2004.07.051, PMID: 15501092

[15] Schilling KG Blaber J Huo Y Newton A Hansen C Nath V. Synthesized b0 for diffusion distortion correction (Synb0-DisCo). Magn Reson Imaging. (2019) 64:62–70. doi: 10.1016/j.mri.2019.05.008, PMID: 31075422 PMC6834894

[16] Baykara E Gesierich B Adam R Tuladhar AM Biesbroek JM Koek HL. A novel imaging marker for small vessel disease based on Skeletonization of white matter tracts and diffusion histograms. Ann Neurol. (2016) 80:581–92. doi: 10.1002/ana.24758, PMID: 27518166

[17] Zhang H Schneider T Wheeler-Kingshott CA Alexander DC. NODDI: practical in vivo neurite orientation dispersion and density imaging of the human brain. NeuroImage. (2012) 61:1000–16. doi: 10.1016/j.neuroimage.2012.03.072, PMID: 22484410

[18] Smith SM Jenkinson M Johansen-Berg H Rueckert D Nichols TE Mackay CE. Tract-based spatial statistics: voxelwise analysis of multi-subject diffusion data. NeuroImage. (2006) 31:1487–505. doi: 10.1016/j.neuroimage.2006.02.024, PMID: 16624579

[19] Rueckert D Sonoda LI Hayes C Hill DL Leach MO Hawkes DJ. Nonrigid registration using free-form deformations: application to breast MR images. IEEE Trans Med Imaging. (1999) 18:712–21. doi: 10.1109/42.796284, PMID: 10534053

[20] Wilson JT Hareendran A Hendry A Potter J Bone I Muir KW. Reliability of the modified Rankin scale across multiple raters: benefits of a structured interview. Stroke. (2005) 36:777–81. doi: 10.1161/01.STR.0000157596.13234.95 PMID: 15718510

[21] Klok FA Boon G Barco S Endres M Geelhoed JJM Knauss S. The post-COVID-19 functional status scale: a tool to measure functional status over time after COVID-19. Eur Respir J. (2020) 56:2001494. doi: 10.1183/13993003.01494-2020, PMID: 32398306 PMC7236834

[22] Delgado DA Lambert BS Boutris N McCulloch PC Robbins AB Moreno MR. Validation of digital visual analog scale pain scoring with a traditional paper-based visual analog scale in adults. J Am Acad Orthop Surg Glob Res Rev. (2018) 2:e088. doi: 10.5435/JAAOSGlobal-D-17-00088, PMID: 30211382 PMC6132313

[23] Soriano JB Murthy S Marshall JC Relan P Diaz JV. A clinical case definition of post-COVID-19 condition by a Delphi consensus. Lancet Infect Dis. (2022) 22:e102–7. doi: 10.1016/S1473-3099(21)00703-9, PMID: 34951953 PMC8691845

[24] Zigmond AS Snaith RP. The hospital anxiety and depression scale. Acta Psychiatr Scand. (1983) 67:361–70. doi: 10.1111/j.1600-0447.1983.tb09716.x 6880820

[25] Smith SM Nichols TE. Threshold-free cluster enhancement: addressing problems of smoothing, threshold dependence and localisation in cluster inference. NeuroImage. (2009) 44:83–98. doi: 10.1016/j.neuroimage.2008.03.061, PMID: 18501637

[26] Benjamini Y Hochberg Y. Controlling the false discovery rate: a practical and powerful approach to multiple testing. J R Stat Soc. (1995) 57:289–300. doi: 10.1111/j.2517-6161.1995.tb02031.x

[27] Petersen M Nägele FL Mayer C Schell M Petersen E Kühn S. Brain imaging and neuropsychological assessment of individuals recovered from a mild to moderate SARS-CoV-2 infection. Proc Natl Acad Sci USA. (2023) 120:e2217232120. doi: 10.1073/pnas.2217232120, PMID: 37220275 PMC10235949

[28] Qin Y Wu J Chen T Li J Zhang G Wu D. Long-term microstructure and cerebral blood flow changes in patients recovered from COVID-19 without neurological manifestations. J Clin Invest. (2021) 131:e147329. doi: 10.1172/JCI147329, PMID: 33630760 PMC8262559

[29] Wenzel J Lampe J Muller-Fielitz H Schuster R Zille M Muller K. The SARS-CoV-2 main protease M(pro) causes microvascular brain pathology by cleaving NEMO in brain endothelial cells. Nat Neurosci. (2021) 24:1522–33. doi: 10.1038/s41593-021-00926-1, PMID: 34675436 PMC8553622

