# Supplementary material for: White matter integrity in hospitalized COVID-19 patients is not associated with short- and long-term clinical outcomes
Source: Front Neurol. 2024 Aug 8;15:1440294. doi: 10.3389/fneur.2024.1440294 (PMC11340528; doi:10.3389/fneur.2024.1440294)
Supplement: Supplementary file 1 [file Table_1.docx]

**Supplemental files to:**

**Title:**

White matter integrity in hospitalized COVID-19 patients is not associated with short- and long-term clinical outcomes

**Supplementary table 1. Ongoing symptoms after COVID-19, reported by the WHO definition of Long COVID**

| Symptom reported | Follow-up 1 (3 months) | Follow-up 2 (12 months) |
| --- | --- | --- |
| Fatigue, n (%) | 20 (44.4) | 14 (31.8) |
| Dyspnea, n (%) | 19 (42.2) | 12 (27.2) |
| Memory loss, n (%) | 3 (6.67) | 5 (11.4) |
| Difficulty finding words, n (%) | 2 (4.44) | 0 (0.0) |
| Myalgia, n (%) | 1 (2.22) | 1 (2.8) |
| Concentration problems, n (%) | 1 (2.22) | 4 (9.1) |
| Tinnitus, n (%) | 1 (2.22) | 0 (0.0) |
| Joint pain, n (%) | 1 (2.22) | 0 (0.0) |
| Numbness fingers, n (%) | 1 (2.22) | 0 (0.0) |
| Dizziness, n (%) | 1 (2.22) | 0 (0.0) |
| Sensory sentivity, n (%) | 0 (0.0) | 1 (2.3) |

**Supplementary table 2. Longitudinal changes of clinical outcomes between 3- and 12-month follow-up in COVID-19 patients**

| Outcomes |  | 3-month  follow-up |  | 12-month follow-up |  |
| --- | --- | --- | --- | --- | --- |
| Cognitive function | *n total* | *Mean (SD)* | *n total* | *Mean (SD)* | *p-value* |
| TICS-M score | 45 | 36.22 (4.16) | 42 | 35.24 (4.66) | 0.301 |
| Functional outcomes | *n total* | *n with outcome*  *(%)* | *n total* | *n with outcome (%)* |  |
| Modified Rankin Scale (mRS)  (reference 0-1)  >2-6, n (%) | 45 | 15 (33.3) | 44 | 16 (36.4) | 0.706 |
| PCFS (reference 0-1)  >2-4, n (%) | 45 | 22 (48.9) | 44 | 22 (50.0) | 0.739 |
| Long COVID | 45 | 28 (62.2) | 44 | 18 (40.9) | ***0.005*** |
| Mood | *n total* | *Median [IQR]* | *n total* | *Median [IQR]* |  |
| *Hospital Anxiety and Depression Scale (HADS)* |  |  |  |  |  |
| HADS - Anxiety, median [IQR] | 45 | 3.0 [1.0, 6.0] | 44 | 3.5 [0.0, 7.0] | 0.814 |
| HADS - Depression, median [IQR] | 45 | 2.0 [1.0, 7.0] | 44 | 3.5 [1.0, 8.0] | 0.357 |

*Abbreviations: COVID-19 = coronavirus disease 2019, TICS-M = Modified Telephone Interview for Cognitive Status, mRS = modified Rankin Scale, PCFS = Post-COVID-19 Functional Status scale, HADS = Hospital Anxiety and Depression Scale*

**Supplementary table 3. Association between Peak width of skeletonized mean diffusivity (PSMD) at baseline and functional clinical outcomes after 3- and 12-month follow-up in COVID-19 patients**

|  | Number of patients (%) | Model 1^a^ |  | Model 2 |  |
| --- | --- | --- | --- | --- | --- |
|  |  | **OR (95% CI)** | **P-value^b^** | **OR (95% CI)** | **P-value** |
| 3-month follow-up | | | | | |
| Modified Rankin Scale (mRS) | | | | | |
| 0-1 | 30 (66.7) | Reference |  | Reference |  |
| 2-6 | 15 (33.3) | 1.29  [0.64 – 2.62] | 0.557 | 3.04  [1.01 – 11.49] | 0.396 |
| Post-COVID-19 Functional Status scale (PCFS) | | | | | |
| 0-1 | 23 (51.1) | Reference |  | Reference |  |
| 2-4 | 22 (48.9) | 1.30  [0.67 – 2.67] | 0.557 | 1.60  [0.59 – 4.91] | 0.664 |
| Long COVID | 28 (62.2) | 1.15  [0.59-2.43] | 0.690 | 2.01  [0.69-7.40] | 0.664 |
| 12-month follow-up |  |  |  |  |  |
| mRS |  |  |  |  |  |
| 0-1 | 28 (63.6) | Reference |  | Reference |  |
| 2-6 | 16 (36.4) | 2.01  [0.98 – 4.85] | 0.360 | 2.32  [0.84 – 7.51] | 0.605 |
| PCFS |  |  |  |  |  |
| 0-1 | 22 (50.0) | Reference |  | Reference |  |
| 2-4 | 22 (50.0) | 1.88  [0.91 – 4.61] | 0.360 | 2.08  [0.76 – 6.82] | 0.664 |
| Long COVID | 18 (40.9) | 1.41  [0.71-2.94] | 0.557 | 2.07  [0.77-6.39] | 0.664 |

*^a^Model 1: adjusted for age, model 2: adjusted for age + WMH volume*

*^b^All P-values are adjusted for multiple comparison using false discovery rate (FDR)*

*Abbreviations: PSMD = Peak width of Skeletonized Mean Diffusivity, mRS = modified Rankin scale, PCFS = Post-COVID-19 Functional Status scale, Long COVID, COVID = coronavirus disease*

**Supplementary table 4. Association between Neurity density index (NDI) at baseline and functional clinical outcomes after three and twelve months of follow-up in COVID-19 patients**

|  | Number of patients (%) | Model 1^a^ |  | Model 2 |  |
| --- | --- | --- | --- | --- | --- |
|  |  | **OR (95% CI)** | **P-value^b^** | **OR (95% CI)** | **P-value** |
| 3-month follow-up | | | | | |
| Modified Rankin Scale (mRS) | | | | | |
| 0-1 | 30 (66.7) | Reference |  | Reference |  |
| 2-6 | 15 (33.3) | 0.74  [0.37 – 1.40] | 0.357 | 0.52  [0.22 – 1.14] | 0.235 |
| Post-COVID-19 Functional Status scale (PCFS) | | | | | |
| 0-1 | 23 (51.1) | Reference |  | Reference |  |
| 2-4 | 22 (48.9) | 0.55  [0.27 – 1.04] | 0.312 | 0.47  [0.20 – 0.97] | 0.235 |
| Long COVID | 28 (62.2) | 0.61  [0.30-1.16] | 0.312 | 0.44  [0.19-0.95] | 0.235 |
| 12-month follow-up |  |  |  |  |  |
| mRS |  |  |  |  |  |
| 0-1 | 28 (63.6) | Reference |  | Reference |  |
| 2-6 | 16 (36.4) | 0.46  [0.21 – 0.91] | 0.185 | 0.46  [0.19 – 0.99] | 0.235 |
| PCFS |  |  |  |  |  |
| 0-1 | 22 (50.0) | Reference |  | Reference |  |
| 2-4 | 22 (50.0) | 0.26  [0.10 – 0.58] | ***0.018*** | 0.23  [0.08 – 0.55] | ***0.018*** |
| Long COVID | 18 (40.9) | 0.55  [0.26-1.05] | 0.312 | 0.45  [0.19-0.95] | 0.235 |

*^a^Model 1: adjusted for age, model 2: adjusted for age + WMH volume*

*^b^All P-values are adjusted for multiple comparison using false discovery rate (FDR)*

*Abbreviations: NDI = neurity density index, mRS = modified Rankin scale, PCFS = Post-COVID-19 Functional Status scale, COVID = coronavirus disease*

**Supplementary Table 5. Associations between Peak width of skeletonized mean diffusivity (PSMD) and Neurity density index (NDI) at baseline and cognition at 3- and 12-month follow-up**

| Diffusion metrics |  | TICS-M after 3 months | | TICS-M after 12 months | |
| --- | --- | --- | --- | --- | --- |
|  |  | *Standardized*  *β [95% CI]* | p*-value^b^* | *Standardized*  *β [95% CI]* | p*-value* |
| PSMD  at baseline | Model 1^a^ | -0.015  [-0.344 – 0.313] | 0.925 | -0.214  [-0.547 – 0.118] | 0.200 |
|  | Model 2 | -0.034  [-0.533 – 0.465] | 0.890 | -0.371  [-0.854 – 0.111] | 0.127 |
| NDI at baseline | Model 1 | -0.020  [0.327 – 0.287] | 0.897 | 0.168  [-0.145 – 0.481] | 0.285 |
|  | Model 2 | -0.027  [-0.389 – 0.335] | 0.881 | 0.189  [-0.174 – 0.553 | 0.298 |

*^a^Model 1: adjusted for age + education (7 levels), model 2: adjusted for age + education (7 levels) + white matter hyperintensities volume*

*^b^All P-values are adjusted for multiple comparison using false discovery rate (FDR)*

*Abbreviations: PSMD = Peak width of skeletonized mean diffusivity, NDI = neurity density index, TICS-M = Modified Telephone Interview for Cognitive Status*

**Supplementary Table 6. Associations between Peak width of skeletonized mean diffusivity (PSMD) and Neurity density index (NDI) at baseline and mood disorder symptoms at 3- and 12-month follow-up**

| Diffusion metrics |  | HADS-Anxiety after 3 months | | HADS-Depression after 3 months | |
| --- | --- | --- | --- | --- | --- |
|  |  | *Standardized*  *β [95% CI]* | p*-value^b^* | *Standardized*  *β [95% CI]* | p*-value* |
| PSMD  at baseline | Model 1^a^ | 0.047  [-0.287 – 0.380] | 0.824 | 0.037  [-0.295 – 0.368] | 0.824 |
|  | Model 2 | 0.356  [-0.136 – 0.848] | 0.339 | 0.140  [-0.365 – 0.644] | 0.773 |
| NDI at baseline | Model 1 | -0.198  [-0.502 – 0.107] | 0.339 | -0.190  [-0.493 – 0.113] | 0.339 |
|  | Model 2 | -0.370  [-0.715 – -0.024 | 0.296 | -0.283  [-0.638 – 0.072] | 0.339 |
| Diffusion metrics | | **HADS-Anxiety after 12 months** | | **HADS- Depression after 12 months** | |
|  |  | *Standardized*  *β [95% CI]* | p*-value* | *Standardized*  *β [95% CI]* | p*-value* |
| PSMD  at baseline | Model 1 | 0.020  [-0.334 – 0.374] | 0.988 | -0.003  [-0.361 – 0.355] | 0.988 |
|  | Model 2 | 0.211  [-0.296 – 0.718] | 0.648 | 0.060  [-0.458 – 0.579] | 0.988 |
| NDI at baseline | Model 1 | -0.206  [-0.523 – 0.112] | 0.396 | -0.267  [-0.583 – 0.050] | 0.256 |
|  | Model 2 | -0.337  [-0.695 – 0.021] | 0.256 | -0.376  [-0.736 – -0.01] | 0.256 |

*^a^Model 1: adjusted for age, model 2: adjusted for age + white matter hyperintensities volume*

*^b^All P-values are adjusted for multiple comparison using false discovery rate (FDR)*

*Abbreviations: PSMD = Peak width of skeletonized mean diffusivity, NDI = neurity density index, HADS = Hospital Anxiety and Depression Scale*

**Supplementary table 7. Association between Peak width of skeletonized mean diffusivity (PSMD) and Neurity density index (NDI) at baseline and Visual Analogue Scale (VAS) at 12-month follow-up**

| Diffusion metrics |  | Visual Analogue Scale (VAS)  after 12 months | |
| --- | --- | --- | --- |
|  |  | *Standardized*  *β [95% CI]* | p*-value^b^* |
| PSMD at baseline | Model 1^a^ | -0.225  [-0.575 – 0.126] | 0.270 |
|  | Model 2 | -0.130  [-0.636 – 0.377] | 0.608 |
| NDI at baseline | Model 1 | 0.344  [0.035 – 0.652] | 0.120 |
|  | Model 2 | 0.319  [-0.039 – 0.677] | 0.158 |

*^a^Model 1: adjusted for age, model 2: adjusted for age + white matter hyperintensities volume*

*^b^All P-values are adjusted for multiple comparison using false discovery rate (FDR)*

*Abbreviations: PSMD = Peak width of skeletonized mean diffusivity, NDI = neurity density index, VAS = Visual Analogue Scale*
